# Supplementary material for: Towards the Identification and Characterization of Putative Adult Human Lens Epithelial Stem Cells
Source: Cells. 2023 Nov 29;12(23):2727. doi: 10.3390/cells12232727 (PMC10706574; doi:10.3390/cells12232727)
Supplement: Supplementary file 1 [file cells-12-02727-s001.zip › cells-2615101-supplementary.pdf]

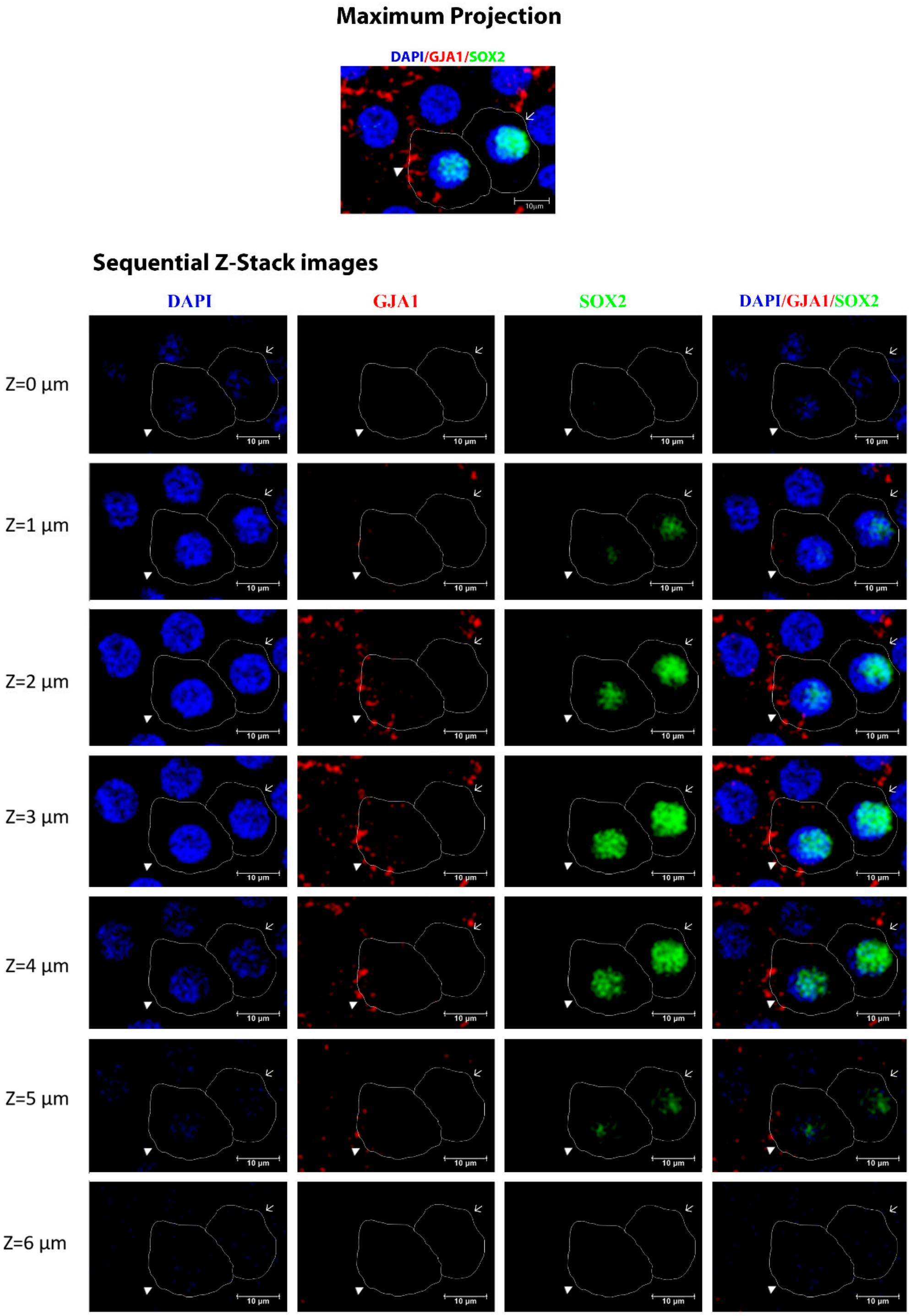

Figure S1. Representative confocal microscopic image of cells in the central zone immunostained for SOX2 (green) and GJA1 (red), counterstained with DAPI (blue). Maximum projection of the z-stack images indicated the presence of two types of SOX2$^+$ cells: SOX2$^+$ GJA1$^-$ cell (arrow) and SOX2$^+$ GJA1$^+$ cell (arrow head). The cells expressing SOX2 but negative for GJA1 membrane expression were defined as putative lens epithelial stem cells. Sequential z-stack images of the same highlighted the membrane expression of GJA1 in the cell highlighted by arrow head. The circle line demarcates the cellular boundary of the highlighted two cells (arrow, arrow head). The speckled plasma membrane expression of GJA1 was observed from z= 1µm to z= 5µm. The plasma membrane expression along the cell boundary was observed (at z= 3µm) where the optical section is through the centre of nucleus (full circle - DAPI positivity).
